# Triggering receptor expressed on myeloid cells-1 in sepsis, and current insights into clinical studies

**DOI:** 10.1186/s13054-024-04798-2

**Published:** 2024-01-09

**Authors:** Vivienne Theobald, Felix Carl Fabian Schmitt, Chiara Simone Middel, Lena Gaissmaier, Thorsten Brenner, Markus Alexander Weigand

**Affiliations:** 1 Department of Anesthesiology, Medical Faculty Heidelberg, Heidelberg University, Im Neuenheimer Feld 672, 69120 Heidelberg, Germany; 2 Department of Anesthesiology and Intensive Care Medicine, University Hospital Essen, University Duisburg-Essen, Essen, Germany

**Keywords:** Sepsis, TREM-1, TREM-1 inhibition, Biomarkers, Targeted therapy, Nangibotide

## Abstract

Triggering receptor expressed on myeloid cells-1 (TREM-1) is a pattern recognition receptor and plays a critical role in the immune response. TREM-1 activation leads to the production and release of proinflammatory cytokines, chemokines, as well as its own expression and circulating levels of the cleaved soluble extracellular portion of TREM-1 (sTREM-1). Because patients with sepsis and septic shock show elevated sTREM-1 levels, TREM-1 has attracted attention as an important contributor to the inadequate immune response in this often-deadly condition. Since 2001, when the first blockade of TREM-1 in sepsis was performed, many potential TREM-1 inhibitors have been established in animal models. However, only one of them, nangibotide, has entered clinical trials, which have yielded promising data for future treatment of sepsis, septic shock, and other inflammatory disease such as COVID-19. This review discusses the TREM-1 pathway and important ligands, and highlights the development of novel inhibitors as well as their clinical potential for targeted treatment of various inflammatory conditions.

## Background

There has been great interest in triggering receptor expressed on myeloid cells-1 (TREM-1) since its discovery in 2000 [1]. Indeed, researchers recently published the interesting results of a phase 2b trial in which patients with sepsis were treated with nangibotide, a TREM-1 inhibitor [2]. TREM-1 belongs to the family of pattern recognition receptors (PRRs) [1, 3–5]. These receptors, which are located intracellularly and on the surface of immune and non-immune cells, recognize microbial/damage-associated molecular patterns (MAMPs/DAMPs) and therefore play a crucial role in host immune defense [6]. Consistently, TREM-1 was first found to be expressed on the surface of monocytes and neutrophils [1], but it is also expressed on non-immune cells like hepatic endothelial cells and gastric epithelial cells. TREM-1 expression on these cell types seems to be dependent on ongoing inflammatory processes [5], highlighting the role of TREM-1 in the immune response [7]. These effects are synergistically enhanced with other well-studied PRRs such as Toll-like receptors (TLRs), especially TLR4 [5], leading to an amplification of the inflammatory response. The role of TREM-1 and other TREM receptors is recently evaluated in more depth in a review by Colonna et al. [8].

Sepsis presents a dysregulated immune response, and TREM-1 has attracted attention as a potential contributor to this condition that often ends in death [9]. The circulating levels of the cleaved soluble extracellular portion of TREM-1 (sTREM-1) are elevated in patients with sepsis and associated with increased mortality in patients with septic shock [10–12]. A study with TREM-1-deficient (*Trem1*^*−/−*^) mice and dextran-sulfate-sodium-induced colitis generated convincing results. The lack of TREM-1 led to a better outcome without negatively impacting pathogen clearance in bacterial and viral infections [13]. In addition, blockade of TREM-1 with the **TREM-1 Fc fusion protein** (**TREM-1/Fc**) was carried out first in 2001. It protected mice against lipopolysaccharide (LPS)-induced shock, as well as microbial sepsis caused by *Escherichia coli* or cecal ligation and puncture (CLP). After the treatment with TREM-1/Fc, mice showed reduced tumor necrosis factor alpha (TNF-α) and interleukin 1β (IL-1β) serum levels and improved survival rates compared with mice that did not receive a specific treatment [3]. Therefore, there have been substantial efforts to develop TREM-1 inhibitors to treat sepsis. LR12, a 12-amino-acid peptide, known as nangibotide (Inotrem, Paris, France), has entered clinical trials. The data are promising for the future therapy of sepsis and septic shock [2, 14], especially considering the current challenges in sepsis therapy. Although its importance is recognized, immune status monitoring is not used in clinical practice as there are currently no direct therapeutic implications. However, current research on adjunctive therapies indicates that there is no therapeutic principle that is suitable for all septic patients, and immune profiling that allows predictive enrichment in current and future treatment scenarios remains a challenge. Furthermore, the use of machine learning and artificial intelligence techniques might improve the development of workable algorithms to guide clinical decisions that make precision medicine for sepsis patients a reality and improve their outcome.

In this review, we critically address the role of TREM-1 as an important PRR in the development and pathogenesis of inflammation and sepsis. We also discuss the latest insights into the safety, efficacy, and therapeutic options of the different TREM-1 inhibitors, including nangibotide.

## TREM-1 and its ligands in the inflammatory state

### Receptor family, structure, isoforms, and pathways

The TREM receptor family—TREM-1, TREM-2, and TREM-3/4/5 (only in mice)—and the immunoreceptor tyrosine-based inhibition motif (ITIM)-bearing TREM-like-transcripts (TLT-1/2/3/4/5) are encoded by genes located on human chromosome 6p21 [15–17].

**TREM-1** is a cell surface receptor belonging to the immunoglobulin superfamily with a related extracellular immunoglobulin-like V-type domain [17]. Besides the membrane-bound isoform (around 30 kDa), a soluble isoform is known, sTREM-1; it contains only the immunoglobulin-like domain, which is essential for antigen recognition and ligand binding [10, 11, 18]. TREM-1 lacks ITIMs in its intracellular domain, and signal propagation is dependent on an adaptor protein, namely DNAX-activating protein of 10 kDa and 12 kDa (DAP10 and DAP12, respectively) [19, 20]. The connection between the two molecules occurs through binding of a positively charged lysine in the receptor and a negatively charged aspartic acid in DAP10/12 [19, 20] and leads to phosphorylation of DAP10/12 and, subsequently, downstream cascades via the activation of spleen tyrosine kinase (Syk) [21]. Multiple signal propagation pathways lead to activation of different molecules and signal cascades involved in inflammatory reactions, leading to enhanced proinflammatory cytokine secretion [8, 12, 19, 21, 22].

### Important ligands for the inflammatory response

The description of TREM-1 ligands is critical for the development of targeted sepsis treatment. Despite the discovery of TREM-1 in 2000 [1], the description of its ligands remains incomplete to date.

One of the first successes in this field was the discovery of **peptidoglycan receptor protein 1** (**PGLYRP1**) [23]. This antimicrobial protein binds to essential factors of the bacterial cell membrane (peptidoglycan and LPS) and induces oxidative stress and lethal cell membrane depolarization [5, 12, 23, 24]. PGLYRP1 activates TREM-1 [18, 25, 26] and binds to sTREM-1 [27].

**LPS** itself is discussed as a potential ligand of TREM-1, because of its role in TREM-1 activation [1, 9]. However, it might be no direct ligand of TREM-1, but interacts indirectly with TREM-1 after binding to TLR4, stimulating the immune system [21].

**High mobility group box 1** (**HMGB1**) was originally described as a DNA-binding protein acting as a cofactor to regulate transcriptional activity [28]. It has subsequently received attention for its role in inflammation. HMGB1 is a DAMP that activates TLRs and is a ligand for the receptor for advanced glycation endproducts (RAGE) [29, 30], which both trigger various cellular cascades leading to inflammatory phenotypes in vitro and in vivo [31]. In 2012, researchers demonstrated in a murine model of hepatocellular carcinoma that HMGB1 released from necrotic hepatocytes bound to TREM-1 with a binding dissociation constant (KD) of 35.4 × 10^–6^ M [12, 32, 33]. In human THP-1 cells, a monocytic cell line, HMGB1 has been shown to increase cytokine production via activating TREM-1.

**Hsp70** isoforms are chaperones, which are involved in a wide variety of cellular protein remodeling, and degradation processes. Therefore, they are crucial for protein homeostasis and have a direct impact on human health [34]. In normal conditions, Hsp70 expression remains low, but it increases strongly in pro-inflammatory states [12, 35]. In 2000, Asea et al. [36] reported that Hsp70 has an additional role as a cytokine and cytokine release driver. This ability increased interest in the role of Hsp70 in inflammation. In 2007, researchers found that Hsp70 along with HMGB1 enhanced the pro-inflammatory response through TREM-1 activation after release from necrotic cells [35]. Recently, researchers showed that Hsp70 can bind to sTREM-1 immobilized on a cyanogen-bromide-activated Sepharose column as well as to TREM-1 on the monocyte surface [12, 37]. This Hsp70/TREM-1 interaction activated interferon (IFN)-γ and TNF-α messenger RNA (mRNA) expression in monocytes and stimulated IL-2 secretion by peripheral blood mononuclear cells [37].

There are high rates of necrosis and apoptosis in sepsis. This leads to a high amount of **extracellular actin,** which can have fatal effects on the organism including the induction of endothelial cell death. These negative consequences underline the importance of the extracellular actin clearance system and its potential role in the pathogenesis of septic shock [38].

In an early study, researchers reported an unknown ligand expressed on platelets that activates TREM-1 in sepsis. Extensive studies using gel analysis of total protein from platelets and **recombinant TREM-1** (**rTREM-1**) revealed that the ligand is actin [39, 40]. The researchers treated murine RAW267.7 cells, a macrophage cell line, with LPS-stimulated platelets and LPS with recombinant actin. They used confocal microscopy to confirm the co-localization of TREM-1 and actin. [12, 40]. Moreover, actin enhanced the release of TNF-α from RAW267.7 cells and peritoneal macrophages via LPS stimulation. The ability of actin to activate TREM-1 could be attenuated by treatment with the TREM-1 inhibitor **LP17**, as well as peritoneal macrophages isolated from *Trem1*^*−/−*^ mice [40]. LP17 was one of the first peptides developed to inhibit TREM-1 through a mechanism analogous to that of decoy receptors [41]. The 17-amino-acid sequence (LQVTDSGLYRCVIYHPP) is derived from a highly conserved sequence between the TREM-1 and TLT-1 extracellular domain in both mice and humans [12, 41].

Another endogenous ligand of TREM-1 was recently described: **extracellular cold-inducible RNA binding protein** (**eCIRP**). This DAMP was originally identified in the blood of patients with hemorrhagic shock and sepsis. In cell models under hypoxic conditions, macrophages could be stimulated by **recombinant CIRP** (**rCIRP**) to release TNF-α and HMGB1 and thereby induce an inflammatory response [42]. Denning et al. [43] demonstrated that murine rCIRP binds to **recombinant murine TREM-1** (**rmTREM-1**) with a KD of 11.7 × 10^–8^ M, which indicates strong affinity. This could also be shown in murine RAW264.7 cells and peritoneal macrophages. To demonstrate that this could have a clinical impact, researchers treated rmCIRP-injected mice with LP17. They observed an attenuated systemic and pulmonary inflammatory response. This study suggests that eCIRP is an endogenous ligand of TREM-1.

### sTREM-1 and its role as a biomarker in sepsis and septic shock

The origin of sTREM-1 is not yet fully understood. Its origin is most likely from proteolytic degradation of TREM-1 by MMPs, such as MMP-9 [44]. Therefore, TREM-1 activation by different triggers such as LPS would be necessary for its cleavage and production [9, 44]. Another relevant source is the translation of a TREM-1 mRNA splice variant lacking the sequence encoding the transmembrane and cytoplasmic regions of TREM-1 [8]. sTREM-1 was first detected in mouse and human serum [41, 45, 46], as well as in bronchoalveolar lavage fluid (BALF) from patients with ventilator-associated pneumonia [47]. In BALF, sTREM-1 is more accurate than any other clinical or laboratory findings to predict the presence of bacterial or fungal infections [8]. High levels of sTREM-1 only occur in infected patients [41, 45, 48], underlining the hypothesis that the origin of sTREM-1 is TREM-1 activation itself. Therefore, sTREM-1 levels indicate TREM-1 pathway activation.

There have been several studies investigating whether sTREM-1 might also be useful as a biomarker of disease severity in different inflammatory settings, especially when an inadequate immune response results in a fatal outcome. Patients with septic shock show extremely high sTREM-1 levels compared with healthy controls (814 pg/mL compared with 1.77–135 pg/mL) [49]. Based on published studies sTREM-1 is only detectable in 19% of healthy controls [1, 3]. In sepsis and septic shock, high sTREM-1 levels are associated with disease severity indicators, including Sequential Organ Failure Assessment (SOFA) score, the Acute Physiology and Chronic Health Evaluation (APACHE) II score, and 28-day mortality [14, 41, 49–51].

In COVID-19, a dysregulated immune state seems to be at least partly responsible for the development of a severe disease course [52]. In COVID-19 [53–56], but also in non-COVID-19 acute respiratory distress syndrome (ARDS) [57, 58], high sTREM-1 levels are associated with disease severity, a prolonged duration of mechanical ventilation, and a poor outcome. Da Silva Neto et al. [54] showed that the best cut-off sTREM-1 level for predicting in-hospital severity for patients with COVID-19 is ≥ 116.5 pg/mL. These inconclusive results concerning optimal sTREM-1 cut-off values underlie the importance of defining robust cut-off values, especially when it comes to the development of therapeutic agents.

In addition to being studied as a prognostic indicator of different inflammatory and non-inflammatory states, sTREM-1 has also been proposed to act as a decoy receptor for TREM-1 [12]. sTREM-1 binds TREM-1 ligands and reduces its activation and the subsequent pro-inflammatory cytokine release [18, 41, 59—61]. First, this theory was tested in vitro. For cloning and expression, the gene for recombinant porcine sTREM-1 was transfected into *E. coli*. Prior to sTREM-1 treatment, porcine alveolar macrophages were stimulated with LPS and then mRNA expression of inflammatory cytokines was evaluated, showing reduced mRNA expression due to sTREM-1 treatment [62]. Interestingly, an in vivo study revealed contrary results. Yang et al. [63] reported worse outcomes in mice infected with *Streptococcus suis*, a bacterium that can rapidly cause streptococcal toxic-shock-like syndrome, and treatment with purified murine sTREM-1. This treatment was associated with increased 7-day mortality, elevated cytokine levels, as well as an increased bacterial load in the blood and peritoneal fluid. Notably, after infection with *S. suis*, mice treated with purified murine sTREM-1 in combination with ampicillin had better outcomes than mice treated with antibiotics alone [64]. Therefore, sTREM-1 agonism might help to decrease some bacterial infections, but it is also responsible for detrimental inflammatory processes leading to acute lung injury [12]. Nevertheless, many of the TREM-1 inhibitory peptides proposed for the treatment of sepsis are based on amino acid sequences found in sTREM-1. This underlines the potential of sTREM-1 as an effective anti-inflammatory mediator [12, 18].

### LR12 and other TREM-1 inhibitors

**Fig. 1:**
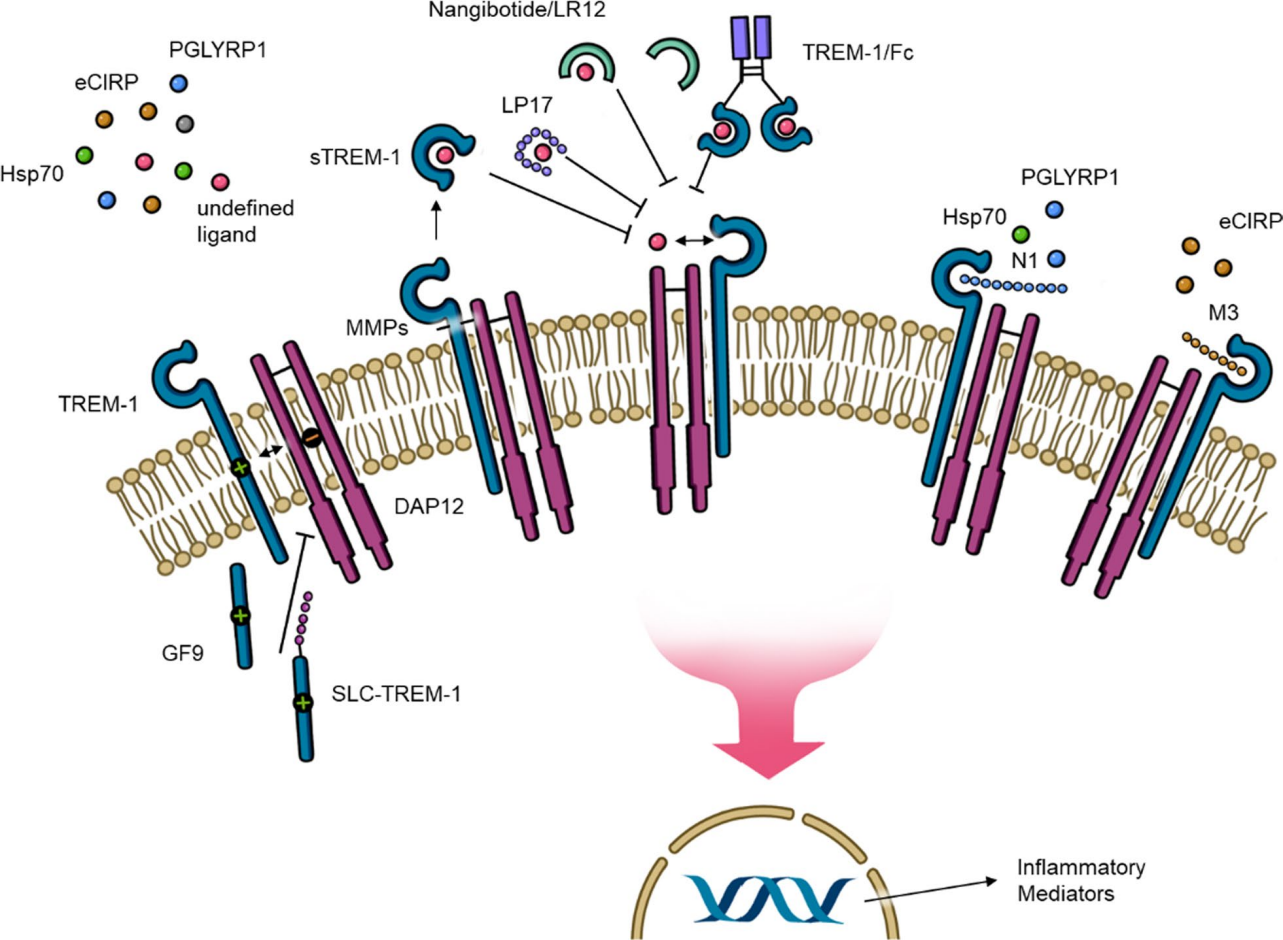
Mechanisms of TREM-1 inhibitors in sepsis TREM-1 activation can be inhibited by different biomolecules: GF9 and SLC-TREM-1 are able to disturb the interaction between TREM-1 and its signaling partner DAP12. LR12/nangibotide, LP17, and TREM-1/Fc fusion protein act as decoy receptors. Decoy receptors are able to recognize and specifically bind ligands of other receptors without activating downstream signaling, so in this case the TREM-1 pathway is blocked. M3, was designed to inhibit a specific ligand of TREM-1, namely eCIRP. N1 is able to block the interaction between PGLYRP1 and TREM-1. Soluble TREM-1 is generated from proteolytic cleavage of membrane bound TREM-1 by matrix metalloproteinases or translation of a TREM-1 mRNA splice variant lacking the sequence encoding the transmembrane and cytoplasmic regions of TREM-1. Circulating soluble TREM-1 competitively binds TREM-1’s ligands and prevents further activation All inhibitors prevent the downstream signaling cascade that upregulates the translation of inflammatory cytokines and TREM-1 receptor. Abbreviations: DAP12 = DNAX-activating protein of 12 kDa protein; eCIRP = extracellular cold-inducible RNA binding protein; Hsp70 = heat shock protein 70; IL = interleukin; MMPs = metalloproteinases; mRNA = messenger RNA; PGLYRP1 = peptidoglycan receptor protein 1; SCL-TREM-1 = TREM-1 sneaking ligand construct; sTREM-1 = soluble triggering receptor expressed on myeloid cells-1; TREM-1 = triggering receptor expressed on myeloid cells-1

LR12 (nangibotide) is a 12-amino-acid peptidic fragment that is derived from TLT-1 [22]. It is the first TREM-1 inhibitor that has entered clinical trials. Nangibotide is chemically synthesized and acts as a ligand-trapping molecule, also called a decoy receptor, that modulates the TREM-1-mediated amplification of inflammatory pathways and the inflammatory response (Fig. 1) [22].

In 2014, Weber et al. [13] generated *Trem1*^*−/−*^ mice. They are viable and fertile, and the hematopoietic compartment shows no signs of alteration, suggesting that serious side effects involving the hematopoietic system are unlikely when receptor blockade is performed. Weber et al. [13] infected *Trem1*^*−/−*^ mice with various bacteria and viruses. They noted lower neutrophilic infiltration as well as decreased morbidity, and the ability to control infection was comparable to the wild type (*Trem1*^+*/*+^) mice [13].

In a minipig model, designed to reflect human physiology more accurately than rodent models [65], researchers induced peritonitis and then either treated or did not treat the animals with LR12. After peritonitis, the mean arterial blood pressure decreased rapidly despite volume resuscitation, and the researchers treated the pigs with a norepinephrine infusion, similarly to septic shock therapy [66]. The LR12-treated pigs required less norepinephrine, indicating a protective effect of the treatment [66]. In addition, thrombopenia occurred less frequently, and in the control group the prothrombin ratio progressively decreased. Despite no differences in the fibrinogen plasma concentration between both groups, this finding suggests an attenuated sepsis-induced coagulopathy through LR12 treatment [66].

Nonhuman primates treated with an intravenous bolus of endotoxin remained normotensive following LR12 treatment. This contrasted with the placebo group, which experienced a 25–40% drop in blood pressure after infusion. In addition, LR12 treatment attenuated endotoxin-induced leukopenia, involving neutrophils, monocytes, and lymphocytes [67, 68]. Moreover, there was a 20–50% reduction in several cytokines in the LR12-treated group [67].

Taken together, these animal studies have demonstrated the impact of TREM-1 on endotoxin-induced endothelial dysfunction, cytokine release and, the inflammatory response. These findings underscore the importance of further investigation of LR12 in humans, particularly in patients with septic shock.

### Additional TREM-1 inhibitors that have not been evaluated in clinical trials

As mentioned previously, LP17 is another TREM-1 inhibitor [12, 41]. Treatment with LP17 could reduce the release of cytokines in LPS-stimulated macrophages [41]. In mice and rats challenged with intraperitoneal LPS, LP17 could improve hemodynamics and survival, and there was reduced release of TNF-α, IL-1β, and IL-6 [12, 41, 69, 70]. Furthermore, LP17 has been shown to improve survival in mice after intravenous administration with *Streptococcus pyogenes *[69]*.* In septic rats induced by LPS injection, LP17 treatment attenuated sepsis severity [70]. There are also interesting results with regard to the treatment of humans. In leukocytes isolated from umbilical cord blood from full term human neonates, LP17 treatment decreased production of TNF-a, IL-6, and IL-8 after exposure to *Escherichia coli* revealing promising results for future therapies of life threating neonatal sepsis [71].

TREM-1/Fc, which is a combined form of the extracellular domain of mouse TREM-1 and the Fc portion of human IgG1, was developed as a TREM-1 decoy receptor [3, 12]. TREM-1/Fc promotes the clearance of TREM-1 ligands from the circulation via Fc receptor–mediated endocytosis [3]. In LPS-, CLP-, and *E. coli*–induced sepsis, mice had improved survival rates due to TREM-1/Fc treatment [3]. These findings were reproduced in two other mouse studies with TREM-1/Fc therapy. Mice showed better survival rates and decreased serum levels of TNF-α, IL-6, and IL-1β [69, 72].

**M3**, a 7-amino-acid sequence (RGFFRGG), was designed to inhibit a specific ligand of TREM-1, namely eCIRP. It was designed based on homology between PGLYRP1 and CIRP [12, 23, 43]. M3 was successfully tested in vitro and in vivo. It decreased TNF-α and IL-6, and improved 7-day survival in mice which were subjected to LPS-induced endotoxemia [43].

Sharapova et al. [73] synthesized a 10-amino-acid inhibitory peptide based on PGLYRP1 to investigate the effects on blocking the interaction between PGLYRP1 and TREM-1. They demonstrated binding of it to sTREM-1, respectively, TREM-1 and named it **N1**. Monocytes and lymphocytes exposed to PGLYRP1/Hsp70 and treated with N1 showed decreased lactate dehydrogenase (LDH) release. In addition, N1-treated cells had lower TNF-α, IFN-γ, IL-1β, and IL-6 mRNA expression after exposure to LPS. In an in vivo model, mice received bronchial instillation of LPS as well as α-galactosylceramide and developed acute lung injury. Afterwards, they were treated with intravenous N1 and were protected against the fatal cytokine storm, denoted by decreased serum levels of INF-γ and IL-4. Additionally, a histological evaluation showed reduced pulmonary inflammation in the treated animals [73].

There are other TREM-1 inhibitors that are not designed to influence the interaction between TREM-1 and its ligands. One of these is **GF9,** a ligand-independent peptide (GLLSKSLVF) derived from the transmembrane region of murine TREM-1. It was designed by Singalov and is able to disturb the interaction between TREM-1 and its signaling partner DAP12 [74, 75]. In LPS-stimulated macrophages and mice, GF9 treatment could decrease serum TNF-α, IL-1β, and IL-6 levels and was associated with higher survival rates in treated animals [12, 75].

**TREM-1 sneaking ligand construct** (**SLC-TREM-1**) is another inhibitor of the interaction between TREM-1 and DAP12. SLC-TREM-1 comprises three modules: an E-selectin targeting domain to bind to the endothelial cell surface, *P. aeruginosa* exotoxin A to facilitate translocation from the endosomal vesicular system into the cytosol, and a 7-amino-acid peptide (LSKSLVF) that contains the interaction site between TREM-1 and its adaptor protein DAP12 [12, 76]. To investigate the in vitro potential of SLC-TREM-1, endothelial cells were stimulated with LPS. They showed decreased TREM-1 expression and activation after treatment with SLC-TREM-1. In an in vivo study, mice subjected to CLP and injected intraperitoneally with SLC-TREM-1 showed improved 10-day survival [76].

**LR17** is a 17-amino-acid peptide close to LP17 [12, 22, 66, 77]. Of note, it is the precursor of LR12. After some promising results concerning sepsis treatment in mice with LR17 [22, 77], it turned out that just 12 amino acids accounted for LR17’s anti-inflammatory effects, and a new peptide containing this sequence (LQEEDAGEYGCM) was designed [9, 12, 22] and is the first TREM-1 inhibitor that has been tested in clinical trials. Besides nangibotide,- several other promising TREM-1 inhibitors (mentioned above) have been identified [12] to treat sepsis. Further studies are needed to investigate their potential for clinical trials (Fig. 1).

### Current insights from clinical trials

In 2018, nangibotide was initially administered in a phase 1 trial to 27 healthy volunteers aged 18 to 45 years at doses of up to 6 mg/kg/h over 7.75 h, preceded by a 15-min loading dose of up to 5 mg/kg. This was safe and well tolerated even at the highest doses, which reached the expected pharmacologically effective dose [78].

The subsequent multicenter, randomized, double-blind, phase 2a trial enrolled patients with septic shock. Treatment with nangibotide or placebo was initiated < 24 h after the onset of shock and continued for up to 5 days at doses of 0.3, 1.0, or 3.0 mg/kg/h. Nangibotide was well tolerated and safe in these patients [14]. There was no difference in inflammatory biomarkers and clinical efficacy between the study groups [12, 14], but in an indirect response model the results revealed a positive correlation between nangibotide concentration and a decrease in the IL-6 production rate [14]. The overall baseline median plasma sTREM-1 level was 433 pg/mL (range: 154–1960 pg/mL). In a subgroup analysis, patients with high levels of sTREM-1 treated with nangibotide had decreased SOFA scores after treatment [12, 14, 65].

In 2019, a double-blind, randomized, placebo-controlled, phase 2b trial (ASTONISH) was started in 42 hospitals in seven countries with medical, surgical, or mixed intensive care units and patients with septic shock. The efficacy and safety of two different nangibotide doses were compared with placebo. Another aim was to identify the optimal treatment population [2]. Like in the preceding phase 2a trial, patients were included in the study within 24 h of vasopressor initiation for the treatment of septic shock. After a loading dose of 5 mg/kg, patients received either nangibotide at 0.3 mg/kg/h (low-dose group) or 1.0 mg/kg/h (high-dose group), or matched placebo. Additionally, at baseline patients were classified according to their sTREM-1 levels as high (≥ 400 pg/mL) or low (< 400 pg/mL). This cut-off was established from observational sepsis studies and observations from the phase 2a trial. The primary endpoint was the mean difference in the total SOFA score from baseline to day 5 in the low-dose and high-dose groups compared with placebo, in the predefined high sTREM-1 (≥ 400 pg/mL) population, and in the overall modified intention-to-treat population. All-cause 28-day mortality, safety, pharmacokinetics, and evaluation of the relationship between TREM-1 activation and treatment response were included in the secondary endpoints. Seventy-one percent of patients were in the a priori–defined high sTREM-1 population [2]. Even though the primary endpoint did not reach significance, this trial revealed promising results. In the high sTREM-1 population, the difference in the SOFA score at day 5 between the high-dose and placebo groups revealed a positive trend, but it was not significant (*p* = 0.104). The same effect could be seen in the overall population (*p* = 0.108). Interestingly, the exploratory analyses revealed clinically relevant benefits and significantly different results among patients with baseline sTREM-1 concentrations > 532 pg/mL (*p* < 0.05). These treatment benefits at increasing sTREM-1 thresholds were seen across all six SOFA subscores [2]. In an exploratory descriptive analysis, the change in the IL-6 levels from baseline to day 2 presented a pattern consistent with the observed clinical effect of a greater reduction at sTREM-1 cut-off values > 500 pg/ml. The study was not powered to detect a significant effect on mortality. The low-dose group had a numerically higher mortality rate, but the authors justified this outcome based on imbalances in the population at baseline [2] (Table 1).

**Table 1:** Important preclinical-, animal developments and clinical trials concerning TREM-1 and TREM-1 inhibitors in infectious diseases, sepsis, and septic shock

Species	Model/condition	Treatment	Effects in the treatment group	References
*Trem1*^*−/−*^ mice	CD4 + T cell and dextran-sodium-sulfate-induced colitis	None	↓ Inflammatory infiltrates ↓ Expression of pro-inflammatory cytokines	[13]
*Trem1*^*−/−*^ mice	Infection with: *Leishmania major* *Legionella pneumophila* Influenza virus	None	↓ Neutrophilic infiltration, lesion size (*L. major*) ↓ Morbidity (influenza virus) = Ability to control infection	[13]
Mice	Sepsis induced by intraperitoneal injection of LPS	LR17: Intraperitoneally injection	↑ Survival ↓ Serum concentrations of TNF-α, IL-6, IL-10,	[22]
Mice	Sepsis induced by CLP	LR17: Intraperitoneally injection after surgery	↑ Survival ↓ TREM-1 expression around 30% ↓ Cytokine concentration in serum, peritoneal and bronchoalveolar fluid, liver and lung ↓ Acute lung injury ↓ Bacteria in spleen and blood	[22]
Minipigs	Fecal peritonitis and hypodynamic septic shock	LR12: Intravenous bolus of 5 mg/kg over 30 min followed by a 1 mg/kg/h (15 mL/h) infusion until the end of the study	↓ Requirement for norepinephrine ↑ Mean arterial pressure, cardiac index, cardiac power index, and SvO2	[66]
Nonhuman primates	Infectious state through intravenous bolus of endotoxin (10 µg/kg)	LR12: Intravenous bolus of 5 mg/kg over 10 min followed by an 8-h continuous intravenous infusion at 1 mg/kg/h (2 mL/h)	Stable blood pressure ↓ Endotoxin-induced leukopenia ↓ 20–50% cytokine plasma concentration (IL-6, IL-8, MCP-1, MIP-1α, MIP-1β, and TNF-α)	[67]
Humans	Healthy volunteers	Nangibotide: Highest dose tested: loading intravenous bolus of 5 mg/kg for 15 min, followed by a maintenance dose of 46.5 mg/kg, administered over 7.75 h (at 6 mg/kg/h)	No accumulation of nangibotide in blood; half-life around 3 min No adverse events related to drug treatment No clinically relevant and significant abnormalities No immunogenicity	[78]
Humans	Septic shock	Nangibotide: Intravenous bolus of 5 mg/kg over 15 min, followed by three different doses for up to 5 days: 0.3 mg/kg/h 1.0 mg/kg/h 3.0 mg/kg/h	↓ SOFA score at day 5 in the high sTREM-1 population (↓) IL-6 plasma concentration in the increasing sTREM-1 level population (based on an indirect response model) No adverse events related to drug treatment No signs of immunogenicity	[14]
Humans	Septic shock	Nangibotide: Intravenous bolus of 5 mg/kg over 15 min, followed by two different doses for a minimum of 3 days and a maximum of 5 days: 0.3 mg/kg/h 1.0 mg/kg/h	↓ SOFA score at day 5 in patients with sTREM-1 > 523 pg/mL ↓ IL-6 plasma concentration in patients with sTREM-1 > 500 pg/mL No adverse events related to drug treatment No clinical signs of immunogenicity	[2]
Monocytes	Infectious state through LPS stimulation	LP17: Cells incubated in LP17 environment at a concentration of 100 ng/ml	↓ Cytokine release (IL-1β, and TNF-α)	[41]
Neonatal leukocytes from full term human neonates	Exposure to: *Escherichia coli*	LP17: Cells incubated in LP17 environment at a concentration of 100 ng/ml for 24 h	↓ Cytokine release (TNF-α, IL-1b, IL-6)	[71]
Mice	Sepsis induced by intraperitoneal injection of LPS	LP17: Intraperitoneally injection of 4 mg/kg for mice	↑ Survival	[70]
Rats	Sepsis induced by CLP	LP17: Intraperitoneally injection of 4 mg/kg for rats 1 h after CLP	↑ Survival ↑ Mean arterial pressure ↑ Aortic and mesenteric blood flow Stable pH ↓ Serum lactate ↓ Cytokine release (TNF- α, IL-1b, IL-6)	[70]
Mice	Infection with: *Streptococcus pyogenes*	LP17: Injection every 24 h intravenously with 200 µl of a 300 µM solution of the synthetically produced conserved extracellular domain LP17 (117 µg)	↑ Survival	[69]
Monocytes	Infectious state through LPS stimulation	TREM-1/Fc treatment	↓ Cytokine release (TNF- α, IL-1b)	[3]
Mice	Sepsis induced by intraperitoneal injection of LPS	TREM-1/Fc treatment	↑ Survival ↓ Serum concentration of TNF-α and IL-1b, ↓ Recruitment of peritoneal macrophages and neutrophils	[3]
Mice	Sepsis induced by CLP	TREM-1/Fc treatment	↑ Survival	[3]
Mice	Infection with: *Escherichia coli*	TREM-1/Fc treatment	↑ Survival	[3]
Mice	Infection with intravenous: *Streptococcus pyogenes*	TREM-1/Fc: Intravenous injection with 5 µg (0.2 mg/kg body weight) 2 h prior and 2 h after bacterial inoculation	↑ Survival ↓ Serum concentration of IL-6 and TNF-α	[69]
Mice	Infection with intraperitoneally: *Pseudomonas aeruginosa*	TREM-1/Fc: Injection of 5 μg 1 h after inoculation	↑ Survival ↓ Serum concentration of IL-1b, TNF-α, MCP-1	[72]
Mice	Sepsis induced by intraperitoneal injection of LPS	M3: Intraperitoneally injection of 10 mg/kg M3	↑ Survival ↓ Serum concentrations of TNF-α, IL-6	[43]
Mice	Sepsis induced by CLP	M3: Intraperitoneally injection of 10 mg/kg M3 during abdominal closure	↑ Survival ↓ Serum concentration of AST, ALT, TNF-α, IL-6 ↓ Acute lung injury	[43]
Monocytes	Infectious state through LPS stimulation	N1: Incubation in a N1 environment	↓ Expression of TNF-α, IFN-γ, IL-1b, and IL-6	[73]
Mice	Sepsis induced by intrabronchial instillation of LPS and α-galactosylceramide	N1: administration of 120 µg N1	↓ Serum concentration INF-γ, and IL-4 ↓ Reduced pulmonary inflammation	[73]
Macrophages	Infectious state through LPS stimulation	GF9: Incubation in a GF9 environment	↓ Cytokine release of TNF-α, IL-1b, IL-6	[75]
Mice	Sepsis induced by intraperitoneal injection of LPS	GF9: Intraperitoneally injection	↑ Survival ↓ Serum concentration of TNF-α, IL-1b, IL-6	[75]
Endothelial cells	Infectious state through LPS stimulation	SLC-TREM-1: Incubation in the presence of 250 or 500 nM SLC-TREM-1	↓ TREM-1 expression ↓ Cytokine release (MCP-1, IL-8)	[76]
Mice	Sepsis induced by CLP	SLC-TREM-1: Intraperitoneally injection of 100 μg SLC-TREM-1 2 h after surgery	↑ 10 day—survival	[﻿^76^]

*ALT* Alanine aminotransferase; *AST* Aspartate aminotransferase; *CD* Cluster of differentiation; *CLP* Cecal ligation and puncture; *FiO2* Fraction of inspired oxygen; *IFN* interferon; *IL* Interleukin; *LPS* Lipopolysaccharide; *MCP* Monocyte chemoattractant protein, *MIP* Macrophage inflammatory protein; *PaO*_*2*_ Partial pressure of arterial oxygen; *SLC-TREM-1* TREM-1 sneaking ligand construct; *SOFA* Sequential organ failure assessment; *sTREM-1* Soluble triggering receptor expressed on myeloid cells-1; *Sv02* Venous oxygen saturation; *TNF* Tumor necrosis factor; *TREM-1* Triggering receptor expressed on myeloid cells-1; *TREM-1/FC*  TREM-1 Fc fusion protein

## Summary and perspectives

TREM-1 is a PRR involved in many inflammatory reactions and contributes to various diseases that are aggravated through an inadequate immune response. Cytokine release leading to the often-described fatal cytokine storm seems to be an important driver in the pathogenesis of septic shock. This life-threatening condition is responsible for an estimated 11 million deaths each year throughout the world [79] and lead to a high personal and financial costs for health care systems [80]. This underlines the importance of developing new therapeutic strategies to improve morbidity and mortality. Currently, except for angiotensin-II and enibarcimab there are no septic shock–specific treatments, of which only angiotensin-II has Food and Drug Administration approval. The current therapeutic options are focused on organ support and control of the infection source [81] Furthermore, the current state of research shows that there are no specific treatment strategies that apply to all septic patients. Immune and non-immune profiling, as well as the use of artificial intelligence, could enable the development of patient-specific therapies in the coming years and improve outcome of patients significantly.

**Fig. 2:**
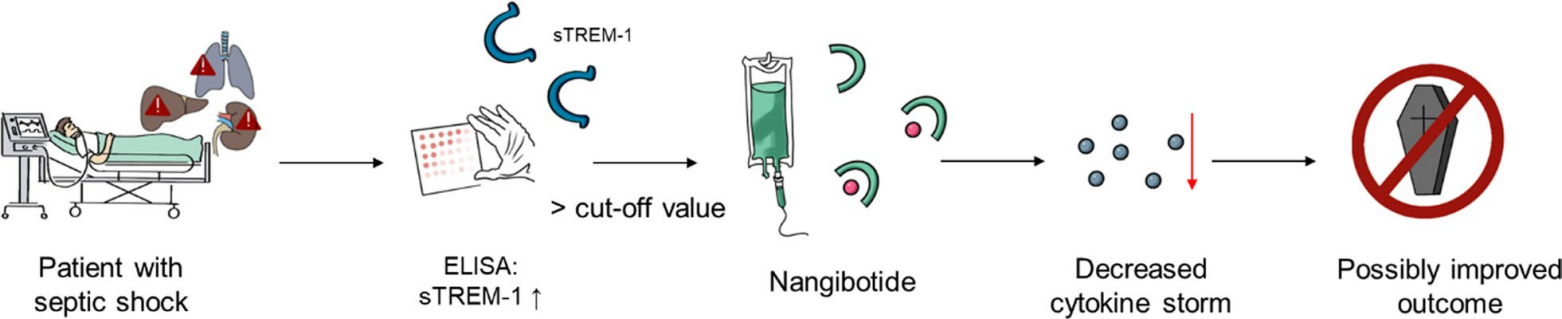
Future principle of personalized and biomarker-driven therapy in sepsis. *The sepsis cascade is a complex process that begins with a suspected infection and leads to a potentially fatal outcome for patients. sTREM-1 might help to detect suitable patients for nangibotide treatment, resulting in a targeted therapy to stop the excessive cytokine release and thereby halt organ dysfunction. Therefore, defining robust cut-off values for sTREM-1 is crucial. Abbreviations: ELISA* = *enzyme-linked immunosorbent assay; sTREM-1* = *soluble triggering receptor expressed on myeloid cells-1; TREM-1* = *triggering receptor expressed on myeloid cells-1*

TREM-1 seems to play an important role in the pathogenesis of sepsis and septic shock, and studies concerning sTREM-1 plasma levels have revealed promising results for sTREM-1 as a biomarker for outcome prediction. sTREM-1 also appears to be relevant to other conditions with an inappropriate immune response, including other infectious and non-infectious disease states. François et al. [2, 14, 82] have investigated nangibotide, a TREM-1 inhibitor in phase 2a and 2b trials in patients with septic shock. Although these studies did not reach the primary endpoint, the authors demonstrated beneficial effects, and there were no adverse events related to treatment. In a subgroup of patients with high plasma sTREM-1 levels, nangibotide treatment led to a significantly better SOFA score at day 5 after septic shock onset, favoring high-dose nangibotide treatment [2, 14]. The change in the SOFA score is a soft outcome parameter: Some authors have described the clinical importance of this change as uncertain [83]. Nevertheless, the authors did provide evidence to support the potential benefit of nangibotide, the treatment appears to be safe, and the early clinical data suggest efficacy. Therefore, nangibotide is an interesting candidate for being tested in a phase 3 trial. However, future studies focusing on the best sTREM-1 cut-off value and the subset of patients with septic shock who might benefit most from nangibotide treatment could be executed before conducting a phase 3 trial. The study could be simplified by using rapid sTREM-1 assays, such as the "near-patient" 1-h assay used by Van Singer et al. [55]. Point-of-care testing could improve early identification of patients at high risk for adverse outcomes and facilitate the judicious use of TREM-1 inhibitors such as nangibotide in the future (Fig. 2).

Although various studies have shown an increase in TREM-1 ligands in septic patients [84–86], to our knowledge there is no data on their change during sepsis. Characterizing this in more detail could help to better coordinate the use of TREM-1 inhibitors during sepsis in clinical practice and improve their beneficial effect in therapeutic strategies.

In addition to clinical research on nagibotide, the other TREM-1 inhibitors should also be further developed and, if possible, brought into the clinical study phase. As it is becoming increasingly clear in current sepsis research that there are different endotypes in septic patients, different adjunctive and targeted therapies are necessary in order to treat patients according to their profile.

## Conclusion

In summary, both sTREM-1 as a biomarker and TREM-1 inhibitors deserve further attention. sTREM-1 as an indicator of TREM-1 activation alongside markedly increased cytokine release could select those patients who would benefit the most from targeted anti-inflammatory or cytokine-reducing therapy—not only in sepsis, but also in other diseases. This view is supported by the growing evidence that IL-6 metabolism can also be positively influenced by TREM-1 inhibitors, like nangibotide. Nevertheless, additional studies to characterize patients more likely to benefit from this adjunctive therapy remain necessary. In addition, despite the exciting results of nangibotide, the other TREM-1 inhibitors should also be further developed and brought into clinical trial phase in order to test and develop other substances beside nangibotide in the treatment of sepsis and septic shock.

## Data Availability

Not applicable.

## Abbreviations

ALT: Alanine aminotransferase
APACHE: Acute physiology and chronic health evaluation
ARDS: Acute respiratory distress syndrome
AST: Aspartate aminotransferase
CD: Cluster of differentiation
CLP: Cecal ligation and puncture
DAP12: DNAX-activating protein of 12 kDa protein
DAMPs: Damage-associated molecular patterns
eCIRP: Extracellular cold-inducible RNA binding protein
ELISA: Enzyme-linked immunosorbent assay
FiO2: Fraction of inspired oxygen
IL: Interleukin
IF: Interferon
ITIM: Immunoreceptor tyrosine-based inhibition motif
HMGB1: High mobility group box 1
Hsp 70: Heat shock protein 70
LPS: Lipopolysaccharide
MAMPs: Microbial-associated molecular patterns
MCP: Monocyte chemoattractant protein
MIP: Macrophage inflammatory protein
MMPs: Metalloproteinases
mRNA: Messenger RNA
PaO2: Partial pressure of arterial oxygen
PGLYRP1: Peptidoglycan receptor protein 1
PRRs: Pattern recognition receptors
RAGE: Receptor for advanced glycation endproducts
rCIRP: Recombinant CIRP
rmTREM-1: Recombinant murine TREM-1
rTREM-1: Recombinant TREM-1
SLC-TREM-1: TREM-1 sneaking ligand construct
SOFA: Sequential organ failure assessment
sTLT: Soluble TREM-like-transcript
sTREM-1: Soluble triggering receptor expressed on myeloid cells-1
Syk: Spleen tyrosine kinase
TLRs: Toll-like receptors
TLT: TREM-like-transcripts
TNF: Tumor necrosis factor
TREM-1: Triggering receptor expressed on myeloid cells-1
TREM-1/FC: TREM-1 Fc fusion protein
